# A Narrative Inquiry Into Problematic Internet Use Among Young Adults: A Narrative Review

**DOI:** 10.7759/cureus.85705

**Published:** 2025-06-10

**Authors:** Alan D Kaye, Rahib K Islam, Victoria T Tong, Marcie L Sorrel, Kaitlyn E Allen, Ivan D Nguyen, Benjamin C Miller, Matthew Sharpe, Shahab Ahmadzadeh, Patricia Griffin, Sahar Shekoohi, Giustino Varrassi

**Affiliations:** 1 Department of Anesthesiology, Louisiana State University Health Sciences Center Shreveport, Shreveport, USA; 2 School of Medicine, LSU Health Sciences Center New Orleans, New Orleans, USA; 3 Department of Anaesthesiology, Louisiana State University Health Sciences Center Shreveport, Shreveport, USA; 4 Departments of Anesthesiology, Louisiana State University Health Sciences Center Shreveport, Shreveport, USA; 5 Pain Medicine, Fondazione Paolo Procacci, Rome, ITA

**Keywords:** addiction, gaming, gaming disorder, problematic internet use, social media addiction, tik tok scrolling

## Abstract

The inescapable influence of the internet and social media has fundamentally transformed human interaction and behavior, leading to a profound digital dependency in a constantly connected contemporary society. This narrative review explores the surge in internet and social media usage globally, driven by the proliferation of new applications and the ubiquity of smart devices. The evolving landscape of digital platforms, exemplified by the rise of TikTok and the multifunctionality of apps like LinkedIn and Notion, blurs the lines between virtual and physical realms, offering diverse avenues for engagement and interaction. Internet addiction, particularly prevalent among young adults, stems from the allure of instant connection and immersive virtual experiences, shaping behavioral patterns and consumption habits from an early age. Beyond individual boundaries, excessive internet usage poses a societal concern with far-reaching consequences, including detrimental effects on mental health, interpersonal relationships, and overall well-being. This review underscores the urgent need to address internet addiction as a pressing public health issue, highlighting its impact on brain regions associated with addiction and emotional regulation. Understanding the psychological impacts of internet and social media usage is paramount in addressing this prevalent problem, with research linking excessive internet use to various psychological struggles, including depression, anxiety disorders, and disordered eating patterns. As internet addiction increasingly affects daily life, this review explores the underlying causes and trends related to emerging applications. It also highlights the importance of promoting digital well-being and addressing the negative impacts of internet addiction on both individuals and communities.

## Introduction and background

In a time when social connection defines our daily existence, the internet and social media have fundamentally altered the nature of human interaction at every level. Every passing day sees the rise of new applications and technology, and with the widespread availability of devices ranging from smartphones to tablets and computers, society finds itself caught in an intricate web of digital dependency [1]. The past decade has witnessed an unprecedented surge in internet and social media usage worldwide. From densely populated urban centers to remote villages, individuals increasingly use digital platforms for a wide range of activities, including communication, entertainment, information sharing, and more [2].

The widespread availability and diverse functionality of smart devices have transformed computers and smartphones from purely personal tools for entertainment or productivity into inherently social devices, often substituting physical social interactions with virtual ones, a phenomenon described as being “alone together” by Sherry Turkle [3]. For instance, TikTok gained prominence in 2019, with its use skyrocketing during the COVID-19 pandemic [4]. Furthermore, the diverse functionality of these platforms extends beyond typical social interactions; even platforms initially designed for professional networking, productivity, or workspace collaboration - such as LinkedIn, Notion, and similar tools - now serve social purposes, highlighting their evolving role in blurring distinctions between virtual and physical interactions [5,6]. Apps like these offer myriad avenues for engagement and interaction, increasingly replacing physical social interactions and blurring the boundaries between virtual and real-world experiences [6].

Internet addiction, often called problematic internet use, refers to the compulsive and excessive use of the internet that leads to significant problems in various areas of life, such as relationships, work, and well-being. Internet addiction is fueled by the promising allure of instant connection and constant engagement. Especially among the younger demographic, the allure of virtual worlds and immersive experiences in video games has become synonymous with modern young adulthood and adolescence. The popular phenomenon often referred to as “iPad Kids” highlights the pervasive influence of digital devices, shaping behavioral patterns and consumption habits from an early age, as discussed in previous literature [7].

The pervasiveness of internet addiction goes beyond individual boundaries, manifesting as a societal concern with far-reaching consequences. From detrimental effects on mental health to the erosion of interpersonal relationships, the adverse consequences of excessive internet usage echo across communities. As technology continues to permeate every aspect of modern life, addressing the public health ramifications of internet addiction becomes essential, calling for concerted efforts to mitigate its impact and promote global digital well-being [8].

To address this public health issue, addiction must be examined through its stages - binge/intoxication, withdrawal/negative affect, and preoccupation/anticipation - each correlating with disruptions in brain regions such as the basal ganglia, amygdala, and prefrontal cortex [9]. The basal ganglia, acting as the brain's reward circuitry, reinforces addictive behaviors through the release of “feel good” neuromodulators like dopamine. Social media features like quick reels and engaging content accelerate addiction cycles, diminishing attention spans, and contributing to emotional dysregulation [10].

Furthermore, it is also important to further understand the research behind the psychological impacts of internet and social media usage, as this will help solve this prevalent problem. Excessive internet use has been associated with several psychological struggles, including difficulties in interpersonal relationships and emotional issues [10]. Longitudinal studies reveal that internet addiction and social media usage are linked to increased depression, anxiety disorders, and dissociative symptoms, while problematic internet use intensifies intrusive thoughts and fear of missing out, exacerbating social anxiety [11,12]. Furthermore, social media usage correlates with body image concerns and disordered eating patterns, especially among women. Sleep patterns are also affected, with internet addiction contributing to shorter sleep duration and further compromising physical and mental health [13].

Amidst this technological revolution, the concern of internet addiction looms large, deeply permeating various aspects of our everyday lives-from the convenience of constant connectivity to its powerful grip on today’s youth. While systematic reviews have addressed isolated facets of excessive internet use-such as its epidemiology or neurobiological underpinnings - no single work has yet woven these threads together with an analysis of today’s rapidly evolving app landscape and its psychosocial drivers. Accordingly, this narrative review synthesizes existing epidemiological data, critically examines the dynamics of emerging digital platforms, and unpacks the behavioral and social factors fueling internet addiction, demonstrating why it has become a pressing public‑health concern.

## Review

Methods

This narrative review was guided by expert judgment rather than a formal systematic protocol. We performed a targeted literature search in PubMed, Embase, and Google Scholar through December 2023 using keywords such as “internet addiction,” “digital dependency,” “social media,” and “app engagement.” We prioritized high-impact empirical studies, key reviews, and seminal conceptual papers, supplemented by manual reference‑list mining. Articles were selected based on relevance to epidemiology, neurobiological mechanisms, psychosocial drivers, or digital‑platform dynamics. Evidence was then synthesized thematically to provide a cohesive exploration of how evolving applications, device usage patterns, and behavioral factors contribute to internet addiction.

Neurobiological mechanisms and impacts of internet addiction

With the onset of advancements and innovation in technology, it has become normalized and even encouraged to maintain a persistent online presence. Internet use is extremely prevalent, therefore not requiring a higher level of impulsivity or risky decision-making that is commonly associated with initial substance use. A vast majority of people utilize digital devices and access the internet without realizing that it can become problematic and progress into an internet gaming disorder or social media addiction through the same mechanisms as other addictive disorders, such as substance use or gambling [14]. Internet gaming disorder and social media addiction are specialized terms referring to problematic internet use on certain interfaces, such as gaming software or social media apps. Maturation of the human brain continues into the early to mid-20s, leaving a large window of increased neuroplasticity during adolescence that is susceptible to remodeling by addiction [15]. Early exposure to the internet and social media can cause profound effects in neurological development, cognitive function, and attention span through the same pathological changes and mechanisms seen in addiction [15].

Addiction is a clinical illness driven by neuronal circuits in the brain that mediate reward and motivation, emotional processing, and executive control [16]. The pathology of addiction is categorized into three stages-binge/intoxication, withdrawal/negative affect, and preoccupation/anticipation [9].

However, addiction is a multifaceted phenomenon and has also been conceptualized through complementary lenses-notably incentive sensitization theory, the allostasis and stress model, the I‑RISA (impaired response inhibition and salience attribution) framework, the impulsivity‑to‑compulsivity transition, and the somatic marker model-each of which emphasizes different mechanistic and individual‑difference factors [9, 16, 17].

The aberrant behavior observed in each stage of addiction is shown to correlate with disruptions in the basal ganglia, amygdala, and prefrontal cortex, respectively. Individuals with addiction may cycle through these stages at variable paces with differing intensities depending on their neurobiology, environment, and individual risk factors. Each addiction cycle results in increased neuroplasticity and brain processes [17].

The basal ganglia are considered the brain’s core ‘reward circuitry,’ releasing dopamine in response to rewarding activities and reinforcing those behaviors [18]. Substance addictions hijack this system with exogenous drugs, producing large, rapid dopamine surges and engaging additional neurotransmitter networks (e.g., GABA, glutamate) in the ventral tegmental area and nucleus accumbens. By contrast, behavioral addictions-such as compulsive gaming or social‑media use-rely on conditioned, endogenous dopamine release (and secondary neuromodulators like endorphins or endocannabinoids) and show a distinct pattern of insular and prefrontal‑striatal plasticity, often with milder tolerance and withdrawal profiles. Over time, both pathways drive compulsive habit formation via the dorsal striatum, but the neurochemical triggers and circuit dynamics differ between substance-based and purely behavioral addictions [19-21].

The use of social media and quick seven-second reels accelerates the addiction cycle, which can result in deleterious effects on one’s attention span [17]. Reels are deliberately curated with engaging clickbait to capture an individual’s attention and keep them entertained for a brief period before they continue to the next reel. Social media clips can provide a rewarding feeling without the effort and commitment that accomplishing a harder task would require [22]. Tolerance to this develops quickly, but users can avoid the withdrawal phase just as quickly by swiping to the next reel. Users can switch through different apps instantaneously, avoiding the negative emotional state caused by an absence of stimuli altogether. Over time, an individual’s attention span will become shorter as they become accustomed to only partaking in activities that provide immediate gratification [23]. The complete avoidance of negative emotional states will contribute to a state of emotional dysregulation [10]. Short attention spans caused by problematic internet usage translate to real-life situations when individuals cannot perform tasks for longer periods of time because they have become accustomed to quicker reward circuits [20]. Therefore, it is important to acknowledge problematic internet usage and its correlation to internet gaming disorder and social media addiction in young adults, where the rate of exposure and use has dramatically risen over the past few decades [15].

Psychological impacts and health consequences of internet and social media addiction

Recent research into the effects of internet and social media use has associated excessive use of these platforms with multiple psychological struggles. Specifically, internet use has been linked to difficulties in interpersonal relationships, depression, anxiety, and struggles with emotional regulation [12]. Longitudinal studies on internet addiction show that individuals with high measures of internet use are more prone to depression, among other mental health issues [24-26]. Further, more severe depression is associated with prolonged internet usage [27]. Another systematic review found positive medium-level associations between internet addiction and depression [28]. In a study of Thai medical students, a significant, mild positive correlation between Internet Addiction Disorder (IAD) and depression was found in medical students, with students having a 1.58 times greater chance of having depression with IAD [29]. Behavioral addiction follows the same ‘three D’s’-dependence, depression, and denial - as substance disorders. Individuals develop a dependence on internet use, experience worsening depressive withdrawal, and often deny the harmful effects-opting to avoid stressful in-person interactions in favor of online social contact [27,30]. Other research suggests that internet addiction increases feelings of loneliness, contributing to the positive relationship between internet use and depression [31].

In another study of addicted and problematic internet users, addicted internet users showed anxiety disorders significantly more often compared to healthy controls, postulated to be due to a lack of in-person positive interpersonal experiences that can escalate previously held self-concept deficits [25]. Continued internet usage is associated with increased degrees of dissociative symptoms, characterized by disruptions in functions of identity, consciousness, memory, and environmental perception that a growing body of literature suggests has a negative influence on the management of psychiatric illnesses such as anxiety [31]. The intensity of problematic Internet use is also associated with “greater intrusive thoughts and fear of missing out” (FOMO) that augment social anxiety [11,12]. In one study of adolescents’ social media activity measured by frequency of account checking and number of accounts held, social media activity was moderately positively related to anxiety, depression, and loneliness, possibly owing to negative comparisons between adolescents’ self-perception to perception of others [32].

Social media use has also been associated with body image concerns and increased levels of disordered eating patterns [33]. Theory proposes that body image ideals presented in online media lead men and, to a greater magnitude, women to increased levels of shame and anxiety about one’s body [32-34]. A study of undergraduate students supports these ideas, as Facebook use positively predicted body consciousness that correlated to greater shame regarding body image 33. Another study of 960 college-aged women showed that women who used Facebook even for 20-minute periods demonstrated a lesser decline in preoccupation with weight than women who did not use Facebook [33,34]. Women with greater disordered eating patterns reported more personal importance of receiving comments and “likes” on their Facebook statuses [34,35]. Alterations in sleep patterns have been found in individuals with internet addiction (IA) who, in one review, were 2.2 times more likely to have sleep problems, specifically shorter sleep duration, than non-addicted individuals, further contributing to negative alterations in physical and mental health [13].

Prevalence and associated outcomes of problematic internet use

Several recent studies have explored the prevalence of problematic internet use (PIU), gaming disorders, or social media addiction, alongside their potential associations with other negative outcomes, including sleep quality, anxiety, depression, stress, negative self-esteem, substance use, and attention deficit hyperactivity disorder (ADHD) symptoms. When examining reports of prevalence, there is considerable variation between studies for what constitutes PIU [36]. However, according to the meta-analysis by Cheng et al. (2021), the pooled social media addiction prevalence from 34,798 respondents across 32 nations was 24% (95% CI (21-28%), p < 0.001) [36].

Sleep Quality

According to the cross-sectional study by Younes et al., 2016, the Insomnia Severity Index (ISI) was completed by participants and compared to the Young Internet Addiction Test (YIAT) results to review the association between sleep quality and PIU among 600 students in medicine, dentistry, or pharmacy programs at Saint Joseph University in Beirut, Lebanon. The YIAT is a self-reported assessment of overall internet use patterns. The ISI evaluates insomnia with a score from 0-28, and clinically significant insomnia is a score of over 14. The mean ISI score in normal internet users versus participants with a potential IA was 8.99 ± 3.65 and 10.89 ± 3.90, respectively, with a p-value of < 0.001 [37].

Anxiety, Depression, and Stress

Younes et al., 2016, also compared results from the Depression Anxiety Stress Scales (DASS) subgroups for stress (S), anxiety (A), and depression (D) to the YIAT results. The mean value of DASS S was 6.50 ± 4.30. and 9.41 ± 4.45 in the normal internet users and potential IA groups, respectively. The DASS A mean value was 4.30 ± 3.53 and 7.09 ± 4.17, respectively, and the DASS D mean value was 4.91 ± 4.19 and 8.02 ± 4.68, respectively. Each of these comparisons was associated with a p-value < 0.0001 37. According to Shannon et al., 2022, the summary metaregression correlation between depressive symptoms, anxiety, or stress and problematic social media use was 0.273 (95% CI [0.215-0.332], p < 0.001), 0.348 (95% CI (0.270-0.426), p <.001), and 0.313 (95% CI (0.203-0.423), p <.001). Each of these measures was associated with heterogeneity between the studies used in the meta-analysis [38].

Negative Self-esteem

Younes et al. (2016) reviewed the association between PIU and negative self-esteem by comparing YIAT results to the Rosenberg Self-Esteem Scale (RSES), reported on a scale of 0-30 where higher scores denote higher self-esteem. The mean RSES values in the normal internet use and PIU groups were 23.05 ± 5.152 and 20.15 ± 5.33, respectively, with a p-value of < 0.0001 [37].

Substance Use

A systematic review of the relationship between PIU, alcohol, and cannabis revealed that there is high variability among studies. The relationship between PIU and alcohol was not consistent [39]. However, the two longitudinal studies with similar methods and sample populations found both a moderate positive association between time spent on the internet and alcohol use [40,41]. In this meta-analysis, there was also a positive association between PIU and cannabis use, however, these results were also highly variable and would require further investigation before definitive comparisons were made [39].

Attention Deficit, Hyperactivity, and Impulsivity

Several studies have shown a positive correlation between PIU and ADHD symptoms. For example, in Kim et al. (2019), the study calculated an odds ratio for developing a smartphone addiction between an ADHD and a non-ADHD group of Korean adolescents. The value, 6.43 with a 95% CI of 4.60-9.00, indicates that ADHD could be a potential risk factor for the development of PIU [42]. However, in the meta-analysis by Augner et al. (2023), the positive correlation between PIU and attention deficit yielded an r = 0.36 (95% CI (-0.04-0.66), p < 0.001). These values for hyperactivity were r = 0.44 (95% CI (0.06-0.70), p < 0.001), and the values for impulsivity were r = 0.41 (95% CI (-0.02-0.71), p < 0.001). Each of these confidence intervals accounts for heterogeneity of the samples. Overall, these values do not sufficiently support the correlation between PIU and both attention deficit and impulsivity, but they do support the correlation with hyperactivity [43].

## Conclusions

Despite our efforts to cover a broad swath of the literature, this narrative review is limited by its non‐systematic methodology, which may introduce selection bias and omit relevant studies. The heterogeneity of diagnostic criteria and outcome measures across included articles also precludes quantitative synthesis or meta‐analysis. Finally, many findings derive from cross‐sectional or short‐term designs, limiting our ability to infer causal mechanisms or long‐term trajectories of IA.

In this narrative review, we set out to bridge several gaps in our understanding of IA by: (1) synthesizing global epidemiological trends, (2) comparing leading neurobiological and behavioral models, and (3) examining how modern app‐design features exploit reward circuitry to drive compulsive use. Our findings indicate that internet and social‐media dependency now affects a substantial share of users worldwide, recruits both classic dopaminergic pathways and stress‐response systems, and is potently reinforced by algorithmic reward schedules and social‐validation loops. However, progress is hindered by inconsistent diagnostic criteria, a lack of longitudinal data on the condition’s natural history, and few intervention studies that integrate policy, design, and therapeutic elements. Moving forward, the field must develop standardized assessment tools, establish long‐term cohorts to track outcomes, and conduct rigorous trials of digital‐wellness interventions to promote sustainable, healthy engagement.
